# Evaluation of the Effectiveness of Mobile App-Based Stress-Management Program: A Randomized Controlled Trial

**DOI:** 10.3390/ijerph16214270

**Published:** 2019-11-03

**Authors:** Won Ju Hwang, Hyun Hee Jo

**Affiliations:** College of Nursing Science, Kyung Hee University, 26 Kyunghee-Daero, Dongdaemun-Gu, Seoul 02447, Korea; hwangwj@khu.ac.kr

**Keywords:** stress management, mental health, mobile health app, mobile mental wellness

## Abstract

Improving mental healthcare using mobile apps might be an effective way to increase interest in mental health and respond to the demand for better psychological health. However, few studies have investigated the effectiveness of app-based stress-management programs. This study aimed to assess the efficacy of an app-based stress-management program. A non-equivalent comparison group pretest-posttest design was used. Participants were randomized into the experimental (*n* = 26) and control (*n* = 30) groups. The experimental group used an application developed for workers for four weeks. The results indicated that stress, emotional labor, self-efficacy, and well-being were significantly different in the experimental group, but the control group’s average scores did not change significantly. On average, the Perceived Stress Scale scores decreased by 1.5 points (*p* = 0.035) and the Korean Occupational Stress Scale scores decreased by 0.87 points (*p* = 0.4). However, depression and anxiety were not significantly different. Emotional labor decreased by 0.16 points (*p* = 0.027), and well-being and self-efficacy mean scores increased by 0.492 (*p* = 0.005) and 0.162 (*p* = 0.025), respectively. These findings support the developed application’s value for promoting mental health and healthy lifestyles. Further research and supplementation are needed for the application’s sustainability.

## 1. Introduction

Demands for high-quality mental health have increased along with interest in mental health [1,2]. In response to this phenomenon, demand for mental healthcare using mobile communications and social networking technologies have increased [3,4]. A survey in 2018 conducted by Korea’s Ministry of Science and Information and Communication Technology regarding internet usage found that 91.0% people aged 6 years or more owned a smartphone, and 89.6% of those aged 4 years or more were smartphone users. Of them, 95.7% used the smartphone more than once per day and, on average, 10.47 times per week.

The very high proportion of smartphone users logically suggests that smartphone applications (apps) for mental healthcare might be a key factor for better mental health [5]. Research on apps that diagnose and monitor mental health conditions and offer counseling and treatment for mental illnesses might provide answers that improve Koreans’ mental health [6]. An epidemiological survey on mental illness found that one-quarter of Korean adults have experienced one or more mental health problems [7]. However, although Koreans experience high stress, they are particularly unaware of their mental health compared to their physical health status [6]. Mental health is an integral and essential component of health. According to the World Health Organization (WHO), health does not merely imply physical conditions; it includes mental health components as well [8]. 

As mental health problems in Korea, such as depression, anxiety, and addiction, have dramatically increased in recent years, so have the costs of treating them, and many programs and plans for mental healthcare are being developed to intercept future problems through preventive efforts and early screening. However, current efforts are limited and not meeting people’s needs [9]. A survey of the residents of Seoul found that 48.3% reported previous experience of mental health problems, such as suicidal ideation, depression, or stress [10]. Most of them usually dealt with the problems without help (27.3%) or with help from family members or friends (47.9%). Almost all the respondents (91.3%) reported that that they recognized the need for help from specialists, but had problems visiting or using the services [11]. Based on these results, improved accessibility to mental healthcare programs is needed in Korea.

As accessibility is a concern, mobile apps might be particularly important for the success of mental healthcare programs. These apps have two important benefits: convenience and usefulness [12,13]. Consumers (patients) and healthcare providers can use apps without the limitations of time or space, and mental health apps could provide healthcare services for many problems, including depression, anxiety, and addiction, based on the WHO’s definition of mental health, which is “the status of being able to deal with not only mental illness but daily stressors as well” [12,14]. These apps also have the benefit of protecting individuals’ personal information [15,16].

Such apps might be effective tools for mental healthcare in Korea [16], but efforts to establish them have been passive [15]. Compared with clients in the United Kingdom and the United States, the number of apps in Korea is remarkably small, and their uses are insufficient [16]. Developing mental health apps and promoting their use should be a new step toward improving mental health. Therefore, the purpose of this study was to develop an app for mental health and assess its feasibility and effectiveness for one month regarding stress management among workers. 

## 2. Materials and Methods 

### 2.1. Study Design

This experimental study employed a nonequivalent comparison group pretest-posttest design to evaluate the effects of the app on employed nurses regarding depression, stress, anxiety, emotional labor, self-efficacy, and well-being. We compared the experimental and control groups on pre- and post-intervention outcome measures. We had the following three hypotheses regarding the experimental compared to the control group after application usage: (1) negative emotional status (stress, anxiety, depression, emotional labor) would decrease in the experimental group, (2) self-efficacy would increase in the experimental group, and (3) well-being would increase in the experimental group.

### 2.2. Subjects and Experimental Procedures 

The participants were 56 nurses employed at college hospitals in Seoul, Korea, and the surrounding metropolitan area. Initially, the experimental and control groups each included 30 subjects. Of the total 71, we randomly assigned 60, except for eight iPhone users and three people who could not run the app. Using the online server provided by the research randomizer (www.randomizer.org) of the Social Psychology Network, subjects were each given a serial number, and then randomly assigned into control or experimental groups. Blinded testing was secured through the different installation of apps in each 30 experimental and 30 control groups.

**Figure 1:**
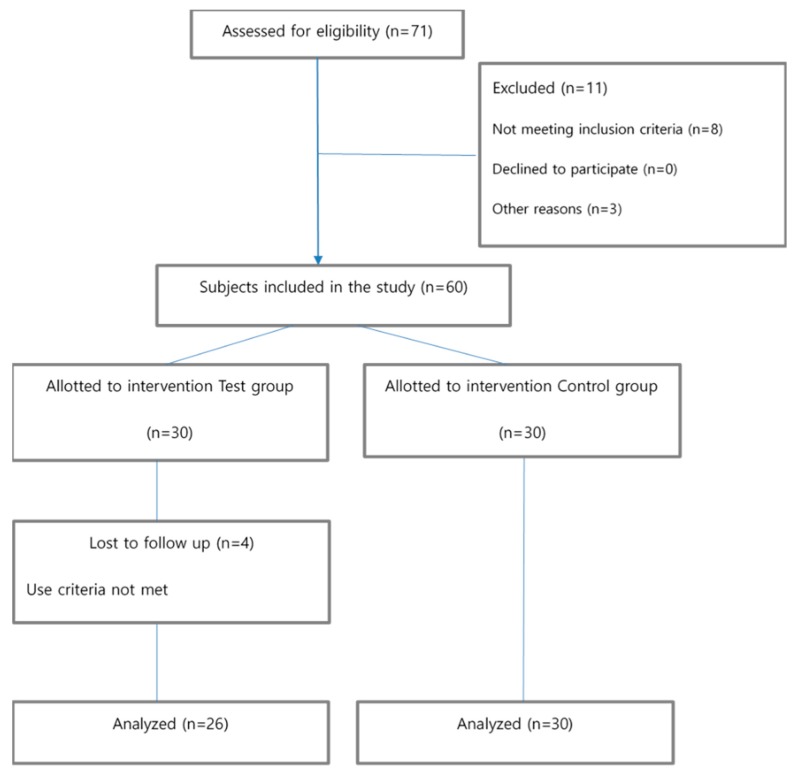
Flowchart of the study design process.

Four subjects in the experimental group stopped using the app and did not complete the final questionnaire, so their questionnaires were excluded from the analysis. The sample size was calculated using G*Power 3.1 [15] program, and 27 participants in both experimental and control groups were required, calculated using the effect size of 0.69 [16], power of 0.80, and confidence level of 0.05 (Figure 1). 

The Institutional Review Board of K University (KHSIRB-18-077(RA)) approved the study’s objectives, methods, and the assurance of subjects’ rights was provided before the study was conducted. Considering the content and ethical issues of this research, participants were fully informed about the study purposes, methods, and expected outcomes. Participants were notified that they could stop the program at any time, and all signed consent forms. The experimental group used the application twice per week for more than 10 min per usage, while the control group was not provided with any such intervention. However, the control group was to use the app after the completion of the experimental study for their personal, and recreational use. 

### 2.3. Outcome Measures

#### 2.3.1. Perceived Stress and Job Stress

A modified version of the Perceived Stress Scale (PSS) developed by Cohen et al. assessed self-awareness of stress (PSS-10) [17]. The PSS-10 includes six items on negative self-awareness and four items on positive self-awareness. The summed scores ranged from zero to 40, and higher scores indicated more self-awareness of stress (Cronbach’s alpha coefficient on the construct was 0.89 in the sample). The second measure of stress was job stress measured using the Korean Occupational Stress Scale (KOSS) developed by Korean Occupational Safety and Health Association [18]. Comprising 26 items rated on a four-point scale, higher scores indicated more stress (Cronbach’s alpha coefficient was 0.86 in the sample).

#### 2.3.2. Depression

Depression was measured following Kroenke et al. [19] using the Patient Health Questionnaire (PHQ)-9. Nine items self-assessed depressive symptoms experienced during the previous two weeks. Response options were rated on a four-point scale, where 0 = *never*, 1 = *sometimes,* 2 = *more than once a week*, and 3 = *almost every day*. The scores on the nine items were summed for total scores that ranged from zero to 27, and higher scores indicated more depressive symptoms (Cronbach’s alpha coefficient was 0.89 in the sample).

#### 2.3.3. Anxiety

The measure of anxiety used was the Generalized Anxiety Disorder (GAD)-7, a self-report tool developed by Spitzer et al. [20] that follows the Diagnostic and Statistical Manual-IV. The seven items included continuous variables and verification questions (the Cronbach’s alpha coefficient was 0.92 in the sample).

#### 2.3.4. Emotional Labor

The Korean-Emotional Labor scale is intended to quantitatively and objectively evaluate the level and intensity of emotional labor and the negative emotional reactions caused by emotional labor, reflecting the specificity of Korea’s organizational culture and service industry. The construct comprised 24 items with response options on a four-point scale and the Cronbach’s alpha coefficient was 0.79 in the study [21]. 

#### 2.3.5. Well-Being

Well-being was measured with the WHO-5 Well-Being Index, which assesses well-being during the past two weeks, with higher scores indicating higher well-being [22,23].

#### 2.3.6. Self-Efficacy

Self-efficacy was assessed based on a Likert scale; higher scores indicated higher self-efficacy, and Cronbach’s alpha coefficient was 0.75 in the sample [24]. 

#### 2.3.7. App Satisfaction 

The level of satisfaction with the app was assessed through a questionnaire given to users that evaluated the pros and cons of using the app, and whether the interest in stress management and the knowledge attitude changed after using the app. 

### 2.4. App Development

**Figure 2:**
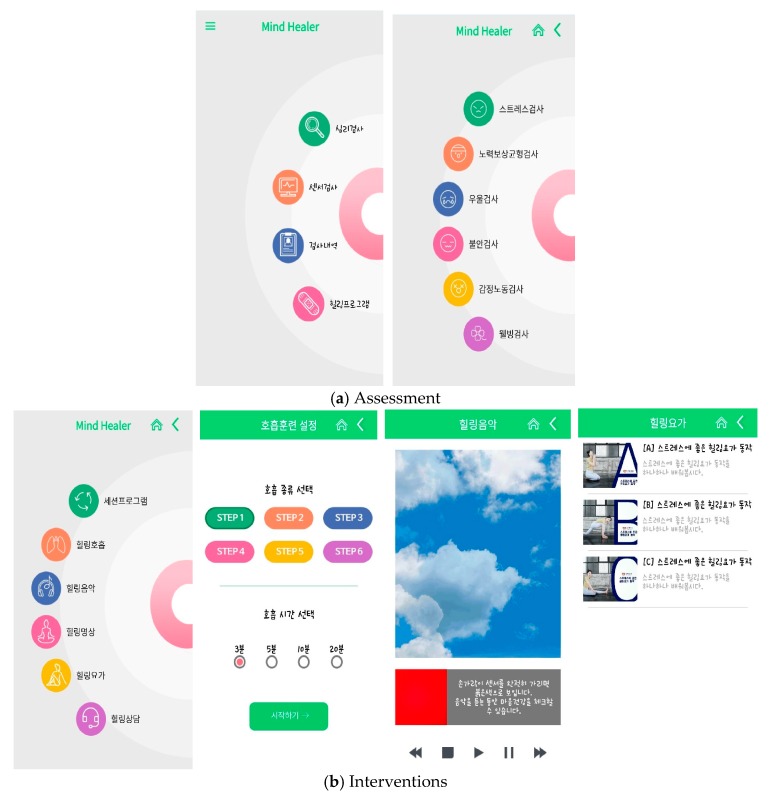
App configuration: (**a**) describe the content of the assessment; (**b**) describe the content of the interventions.

The app that was tested in this study was developed by systematic literature review and the need assessment surveys published in September through December 2018. The app consisted of music focused on healing, meditation, breathing methods, and yoga intervention, including health information for mental health care every week (diet, benefits of exercise, etc.) (Figure 2).

### 2.5. Analytical Methods

This study was an experiment intended to assess the effects of the developed app. After installation of the smartphones, the subjects in the experimental group were asked to freely use the app for four weeks, more than twice per week, and for at least 10 min per usage. SPSS 21.0 for Windows (IBM Corp, Armonk, NY, USA) was used to analyze the data. The general characteristics of experimental and control groups were calculated as frequency, percentages, means, and standard deviations. The homogeneity test of the dependent variable was analyzed using *t*-test. To compare the effectiveness of the apps in the experimental and control groups, the analysis was conducted with paired *t*-test, and repeated measure analysis of variance (ANOVA). App satisfaction was analyzed as frequency and percentage and contents as well.

## 3. Results

The homogeneity test results are shown in Table 1 and Table 2. Table 1 describes the subjects’ personal characteristics (sex, age, marital status, educational attainment, and monthly income) and Table 2 indicates no significant differences between the experimental and control groups regarding the key variables in the study, verifying their homogeneity on those variables. The mean perceived stress score was 20.0 ± 4.18 in the experimental group and 18.6 ± 3.72 in the control group, and mean depression (PHQ-9) was 7.11 ± 4.49 in the experimental and 7.53 ± 6.14 in the control group, which was considered low. Mean anxiety (GAD-7) in the experimental group was 4.26 ± 3.42, which was slightly higher in the control group (4.40 ± 4.34); both means were considered low. Mean well-being was 2.06 ± 0.98 and 2.20 ± 0.95 in the experimental and control groups, respectively.

**Figure 3:**
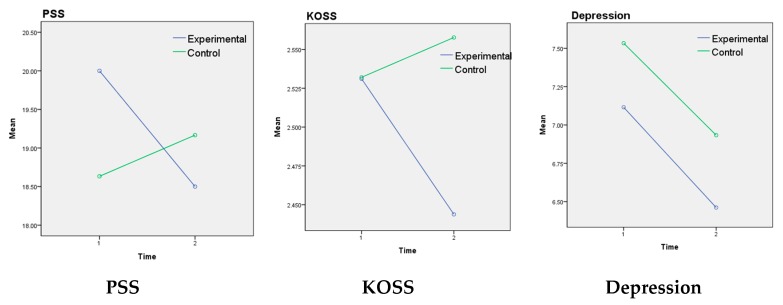

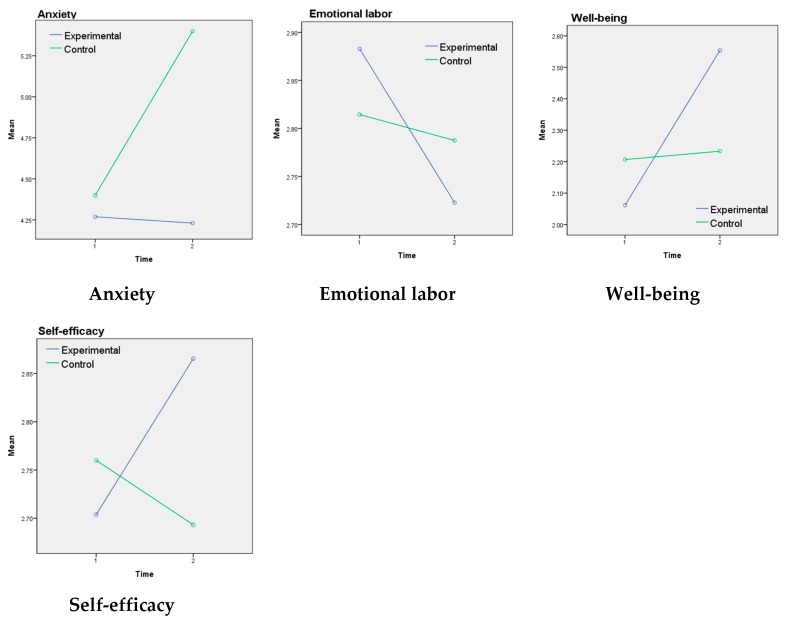
Differences between the experimental and control groups.

Table 3 and Figure 3 showed the results of the use of the app is experimental and control groups. Both measures of stress, PSS and KOSS were significantly different in the experimental group after the four-week treatment period. Specifically, PSS decreased by 1.50 points on average (*p* = 0.035), repeated measures ANOVA showed a significant difference in the interaction between group and time (F = 3.33, *p* = 0.037). KOSS decreased by 0.87 points (*p* = 0.04). Repeated measures ANOVA also revealed a significant difference in the interaction between group and time (F = 3.97, *p =* 0.050). However, although mean depression and anxiety scores decreased, those differences were not statistically significant for either group. Emotional labor showed a great difference before and after the experimental treatment (*p* = 0.027) with a decrease of 0.16 points. Repeated measures of variance analysis of well-being showed no significant difference between the two groups (F = 0.133, *p* = 0.717), but there was a significant difference between the two-time points (F = 5.06, *p* = 0.029). Repeated measures ANOVA showed a significant difference in the interaction between group and time (F = 4.07, *p* = 0.048). The difference before and after the intervention in the experimental group increased significantly by 0.49 points (*p* = 0.005). There was a significant difference in self-efficacy in the interaction between group and time (F = 5.65, *p* = 0.021): the experimental group was significantly higher in the former than 0.16 points after the use of app (*p* = 0.025).

Table 4 reports the results on the users’ satisfaction and experience with the app. It indicates that about 69.2% users were satisfied and found it useful for stress management. About 57.7% of the users reported that, by using the app, their stress awareness increased and 61.5% of the users reported increased motivation to seek treatment. In addition, knowledge about treating stress (46.2%) and attitude (53.8%) improved, and 46.1% users reported changed behaviors. Additionally, it was reported that the app was effective in checking users’ stress levels and mental health promotion in response to their satisfaction and experience with the app.

## 4. Discussion

The purpose of this randomized control trial was to assess the effectiveness of a mental healthcare app developed for workers to self-manage stress and use mental healthcare programs such as meditation, sound, and yoga through the app. The study found that the app for stress management effectively improved mental health. At posttest, the experimental group’s stress, depression, anxiety, and emotional labor improved. Moreover, the positive index of their well-being and self-efficacy level increased. An interest in mobile mental health apps for well-being is growing in light of the many mobile physical healthcare apps becoming available [25]. 

First, participants tried to check the effect of the app by distinguishing the work stress from the perceived stress as they work in many environments, while performing complex and diverse roles to identify the perceived stress and job stress separately. Stress is a psychological and physical state of strength that a person experiences when he or she is in a difficult environment, and when stress accumulates, it triggers psychological tension, anxiety, and depression, and is physically related to cardiovascular disease and sleep disorders [26,27,28]. Some real-time mobile apps for stress and anxiety management have been effective in many ways [25,29,30,31]. This study measured users’ perceived stress, job stress, anxiety, depression, and emotional labor with psychological tests administered on the mobile app. Then, real-time observations of change and mental health were possible [32]. Both the perceived stress (PSS) and job stress significantly reduced in the experimental group. This is useful for stress management and perceived job stress. Ahtinen et al. pointed out that an app positively influenced the treatment of stress and improved the quality of life, which was similar to our overall results [33]. Zeiden et al. argued that short and careful intervention in stressful situations is effective in reducing tension, fatigue, anxiety, and heart rate. [34]. Thus, our evidence supports previous studies that found that apps have potential for stress relief regarding prevention of and immediate responses to stress as the knowledge of app users increases. However, the sample size in this study was small (*N* = 56) and the intervention period was also short (four weeks), so results should be interpreted with care. Also, no statistically significant effects were found on depression and anxiety for both experimental and control groups.

Second, the depression and anxiety level of the participants decreased. However, the decreases in both depression and anxiety have not been found to be significant. This is because the experimental and control groups were in the normal range before and after the treatment and the short study period of four weeks. However, the stress exposure model, the perspective that stress causes depression, and the situations in which the individual’s depressive symptoms and behaviors become stressful, are self-perpetuating, which in turn causes depression [27]. In both stress-generation models [35], the increase in stress is related to the increase in depression, so it is important to manage them mutually. Anxiety is also closely related to how stress is managed [36]. However, the intervention provided by the stress management app lacks positive stress coping methods to reduce anxiety and thus requires continuous development.

Our study found that the app did not have a significant influence on mean depression or anxiety scores, but this result might be related to the fact that depression and anxiety scores were low before the four-week treatment period, so little improvement was possible. However, some previous studies have found that results using the PHQ-9 to measure depression administered via mobile apps were more convenient for analysis than using the conventional PHQ-9 administration, and they were better at evaluating suicide or attempted suicide [37]. Mobile mental health apps might be a very effective approach because they help identify people who need mental health services while protecting personal data [38]. However, Huguet et al. pointed out problems with treating depression using just remote treatment with mobile apps [39]. Therefore, a variety of approaches and interventions are needed, such as nursing, other healthcare services, psychology, and media and app development experts, to improve access to mental healthcare management using apps.

Third, Pisaniello et al. found that emotional labor had a strong influence on health at the workplace and an impact on job satisfaction [40]. Emotional labor might relate to stress and lead to poor well-being and health conditions. From this perspective, using the app might help to improve and maintain mental health and emotional labor, which might lead, in turn, to improved self-efficacy and well-being. 

Fourth, this study found that using the app had positive influences on well-being and self-efficacy. The previous study found that happiness was improved after using the mobile app program, which possibly improved well-being [41]. This finding is similar to our study’s finding that meditation and sound applied using the app helped to decrease depression and/or anxiety and improve well-being. 

In addition, Lee and Yu showed that job stress had an inverse correlation with self-efficacy, and higher job stress showed lower self-efficacy [42,43]. Results of this study showed, that the use of the app reduces stress and increases self-efficacy, and the continuous use of the app is expected to reinforce it. Moreover, when a user’s reasons for using the app were clear, satisfaction with and the amount of usage were significantly higher than when a user was unsure, and that aspect of the study should be emphasized in future research.

Finally, to routinely use mobile apps for stress management requires considerable effort. The data on satisfaction with app usage was obtained through both questionnaire surveys and phone interviews concurrently. It revealed that about 69.2% of users were satisfied with the study’s app. Participants were positive that “the app is always available and easy to use”. Additionally, it was reported that “It is effective to reduce stress with short, and easy programs”. Some previous studies found that using health-related apps directly related to satisfaction with and continued use of the apps [44,45]. In other words, individuals who are confident that using a healthcare app will improve their health management, and know and understand the mental healthcare information offered by an app, consider the app to be a useful tool. This suggests that the increase in self-efficacy found in this study might be accomplished simply by providing a user-friendly app, which might motivate increased stress management and changed behaviors. Lee et al. found that the app would be useful when the users find it enjoyable, which would be likely to increase the amount of usage [46]. Two-way communication channels and real-time responses were influential factors. To obtain individuals’ sustained uses of this app, it must be modified and supplemented in ways intended to increase user satisfaction and intention to use. Two-way communications and additional options for users’ abilities to independently set and reach goals should be incorporated into the app and be frequently studied, evaluated, and modified.

There are some limitations. The developed apps for healthcare, particularly mental healthcare, have not kept up with the rates of smartphone and mobile app usage. Despite these positive results, as this is a preliminary study, it has some limitations including small sample size and short intervention duration. We suggest an extension of this study with a wider sample. Future studies should incorporate continuous counseling and provision of health information with the app’s current functions, and more factors should be analyzed. 

This study was conducted as a pilot test to confirm results by applying the developed app. Although the number of subjects and the participating professions were limited, the effect size of the stress-management app was 1.303, which was high. Based on this work, the research to apply the improved app based on feedback needs to expand the number of subjects and professions and extend the research period. It is also necessary to follow the guidelines of the Consolidated Standards of Reporting Trials to add sophistication to the study and reflect intent-to-treat analysis.

## 5. Conclusions

In conclusion, this study aimed to determine the effectiveness of a mental healthcare app for stress management. Using a pre- and post-test experimental design, the developed app was applied for four weeks and found to significantly contribute lower depression, stress, emotional labor, and anxiety, and increase self-efficacy and well-being in an experimental group of employed nurses. An increase in the number of developed apps for healthcare, particularly mental healthcare, is necessary to keep up with the rates of smartphone and mobile app use. Despite the study’s positive results, its small sample size and short intervention period are its limitations. Future research should include a wider sample, continuous counseling and health information provision via the app’s current functions, and the analysis of more factors. These steps ultimately might result in a unique mobile mental healthcare app that reliably contributes to healthier lifestyles and better mental health. 

## Figures and Tables

**Table 1:** Homogeneity tests of personal characteristics (*N* = 56).

Variable	Category	*N* (%)	Experimental Group (*N* = 26)	Control Group (*N* = 30)	χ^2^/Z	*p*-Value
**Sex**	Male	3 (5.4)	1	2	−0.463	0.643
	Female	53 (94.6)	25	28		
**Age** (years)	<30 ^a^	14 (25)	4	10	1.307	0.279
	31–40 ^a^	30 (53.5)	15	15		
	>40 ^a^	12 (21.5)	7	5		
**Marital status**	Unmarried	27 (48.2)	11	16	−0.816	0.414
	Married	29 (51.8)	15	14		
**Educational attainment**	College	26 (46.4)	16	18	−0.117	0.907
	>College	30 (53.6)	10	12		
**Income** (won millions)	<200 ^a^	4 (7.2)	2	2	0.158	0.854
	201–400 ^a^	26 (46.4)	11	15		
	>400 ^a^	26 (46.4)	13	13		

^a^ Scheffe test result.

**Table 2:** The homogeneity of the measured variable (*N* = 56).

Variable		Experimental Group (*N* = 26) M ± SD	Control Group (*N* = 30) M ± SD	*Z*-Value	*p*-Value
**Perceived health**	Physical health	3.27 ± 0.72	3.23 ± 0.82	−0.080	0.936
	Mental health	3.42 ± 0.76	3.30 ± 0.75	0.493	0.622
**Stress**	PSS ^1^	20.00 ± 4.18	18.63 ± 3.72	−0.933	0.351
	KOSS ^2^	2.53 ± 0.31	2.53 ± 0.27	−0.198	0.843
**Depression**		7.11 ± 4.49	7.53 ± 6.14	−0.025	0.980
**Anxiety**		4.26 ± 3.42	4.40 ± 4.34	−0.306	0.759
**Emotional labor**		2.88 ± 0.41	2.81 ± 0.32	−0.617	0.537
**Well-being**		2.06 ± 0.98	2.20 ± 0.95	−0.396	0.692
**Self-efficacy**		2.70 ± 0.45	2.76 ± 0.40	−0.058	0.954

^1^ PSS: Perceived Stress Scale; ^2^ KOSS: Korean Occupational Stress Scale.

**Table 3:** The difference of the experimental and control group results.

Variable		Group	Pretest EM ^1^ ± SE	Posttest EM ± SE	Source	F-Value	*p*-Value
Stress	PSS	Experimental	20.00 ± 0.77	18.50 ± 0.70	Group Time Group*Time	0.172 0.754 3.33	0.680 0.389 0.037 ^†^
			md ^2^ = 1.50, *p* = 0.035				
		Control	18.63 ± 0.72	19.16 ± 0.65			
			md = −0.533, *p* = 0.485				
	KOSS	Experimental	2.53 ± 0.05	2.44 ± 0.05	Group Time Group*Time	0.645 1.18 3.97	0.425 0.282 0.050
			md = 0.087, *p* = 0.040				
		Control	2.53 ± 0.05	2.55 ± 0.05			
			md = −0.026, *p* = 0.51				
Depression		Experimental	7.11 ± 1.06	6.46 ± 0.98	Group Time Group*Time	0.117 1.440 0.003	0.734 0.235 0.959
			md = 0.654, *p* = 0.396				
		Control	7.53 ± 0.99	6.93 ± 0.91			
			md = 0.600, *p* = 0.403				
Anxiety		Experimental	4.26 ± 0.77	4.23 ± 0.86	Group Time Group*Time	0.412 1.040 1.212	0.524 0.312 0.276
			md = 0.038, *p* = 0.966				
		Control	4.40 ± 0.72	5.40 ± 0.80			
			md = −1.00, *p* = 0.126				
Emotional labor		Experimental	2.88 ± 0.07	2.72 ± 0.08	Group Time Group*Time	0.000 3.76 1.91	0.980 0.580 0.173
			md = 0.160, *p* = 0.027				
		Control	2.81 ± 0.06	2.78 ± 0.07			
			md = 0.027, *p* = 0.683				
Well-being		Experimental	2.06 ± 0.19	2.55 ± 0.20	Group Time Group*Time	0.133 5.06 4.07	0.717 0.029 0.048
			md = −0.492, *p* = 0.005				
		Control	2.20 ± 0.17	2.23 ± 0.18			
			md = −0.027, *p* = 0.866				
Self-efficacy		Experimental	2.70 ± 0.08	2.86 ± 0.08	Group Time Group*Time	0.094 0.977 5.65	0.000 0.327 0.021
			md = −0.162, *p* = 0.025				
		Control	2.76 ± 0.07	2.69 ± 0.08			
			md = 0.067, *p* = 0.313				

^1^ Estimated mean; ^2^ mean difference; ^†^ one-sided test.

**Table 4:** The users’ satisfaction and experience with the app (*N* = 26).

Satisfaction Item		*N* (%)	Users’ Experience
Satisfaction with the app	Dissatisfied	8 (30.8)	Stress index objectively verified Enhanced mental health Various programs Easy accessibility Less use time
	Satisfied	18 (69.2)	
Does this app raise your stress management awareness?	Decreased	0 (0)	
	No change	11(42.3)	
	Improved	15 (57.7)	
Does this app increase your stress management knowledge?	Decreased	2 (7.7)	
	No change	12 (46.2)	
	Improved	12 (46.2)	
Does this app improve your attitude toward stress management?	Decreased	2 (7.7)	
	No change	10 (38.5)	
	Improved	14 (53.8)	
Does this app provide stress motivation for management?	Decreased	0 (0)	
	No change	10 (38.5)	
	Improved	16 (61.5)	
Does this app bring behavioral change in stress management?	Decreased	4 (15.4)	
	No change	10 (38.5)	
	Improved	12 (46.1)	
